# Photoinhibition of photosystem I under high light in the shade-established tropical tree species *Psychotria rubra*

**DOI:** 10.3389/fpls.2015.00801

**Published:** 2015-09-29

**Authors:** Wei Huang, Shi-Bao Zhang, Jiao-Lin Zhang, Hong Hu

**Affiliations:** ^1^Key Laboratory of Tropical Forest Ecology, Xishuangbanna Tropical Botanical Garden, Chinese Academy of Sciences, Mengla, China; ^2^Key Laboratory of Economic Plants and Biotechnology, Kunming Institute of Botany, Chinese Academy of Sciences, Kunming, China

**Keywords:** cyclic electron flow, high light, photoinhibition, photosystem I, shade-established species

## Abstract

The photosynthetic sensitivity to high light differs among understory plants of shade- and sun- established tree species. Shade-established tree species are sensitive to high light but the underlying photosynthetic mechanism has not been fully resolved. In the present study, we examined the responses of photosystem I (PSI) and photosystem II (PSII) to high light in shade leaves of a shade-established tree species *Psychotria rubra* and a sun-established tree species *Pometia tomentosa*. After exposure to 2000 μmol photons m^–2^ s^–1^ for 2 h, the maximum photo-oxidizable P700 (*P_m_*) decreased by 40 and 9% in *P. rubra* and *P. tomentosa*, respectively. These results indicate that the shade-established species *P. rubra* is incapable of protecting PSI under high light. Strong photoinhibition of PSII under high light led to large depression of electron transfer from PSII to PSI and then prevented further photodamage to PSI. During the high light treatment of 2000 μmol photons m^–2^ s^–1^, PSI photoinhibition in *P. rubra* was accompanied with high levels of cyclic electron flow (CEF) and P700 oxidation ratio. Therefore, we propose that PSI photoinhibition under high light in *P. rubra* is dependent on electron transfer from PSII to PSI, and CEF is unlikely to play a major role in photoprotection for PSI in *P. rubra*. These findings suggest that photoinhibition of PSI is another important mechanism underlying why shade-established species cannot survive under high light.

## Introduction

Under high light condition, excess absorbed light energy can induce photoinhibition (Powles, 1984; Barber and Andersson, 1992; Aro et al., 1993a,b; Takahashi et al., 2009; Takahashi and Badger, 2011), especially in shade leaves (Aro et al., 1993b; Kitao et al., 2000; Barth et al., 2001; Krause et al., 2004). In the understory of tropical rain forests, leaves grown in light condition of deep shade (<5% of full sunlight). When canopy gaps are created by tree fall, shade leaves may be exposed to direct sunlight for several hours in a day. High light induced a large decrease in PSII activity in shade leaves of pioneer and late-succession tree species (Kitao et al., 2000; Barth et al., 2001; Krause et al., 2004), while photosystem I (PSI) activity was usually unaffected in these shade leaves (Barth et al., 2001). However, it is unclear whether PSI activity is susceptible to high light in shade-established tree species.

Previous studies have indicated that PSI photoinhibition mainly occurs in chilling-sensitive species including cucumber and *Arabidopsis thaliana* when exposed to chilling-light stress (Havaux and Davaud, 1994; Terashima et al., 1994; Sonoike, 1995; Barth and Krause, 1999; Zhang and Scheller, 2004). Photoinhibition of PSI is mainly caused by the oxidation of hydroxyl radicals that are generated by a reaction between reduced iron–sulfur centers and hydroxyl peroxide (Sonoike, 1995, 2011). Therefore, over-reduction of PSI acceptor side and production of hydroxyl peroxide are the two main causes of PSI photoinhibition (Sonoike et al., 1997; Munekage et al., 2002). Plants have several mechanisms to protect PSI against photoinhibition, such as anti-oxidative scavenging system (Hwang et al., 2004) and cyclic electron flow (CEF; Munekage et al., 2002). CEF can protect PSI activity by increasing the P700 oxidation ratio (Munekage et al., 2002, 2004). P700^+^ can harmlessly dissipate excess light energy as heat and protect PSI activity (Nuijs et al., 1986). Although wild type of *Arabidopsis thaliana* and cucumber have CEF activity (Kim et al., 2001; Munekage et al., 2004), they display PSI photoinhibition when illuminated at chilling temperature (Terashima et al., 1994; Kudoh and Sonoike, 2002; Zhang and Scheller, 2004). These results suggest that PSI photoinhibition cannot be wholly prevented by the activation of CEF under extreme environmental stresses. Since high light can cause an over-reduction of photosynthetic electron transport chain and production of a large amount of hydroxyl peroxide in the acceptor side of PSI (Murata et al., 2007), it is speculated that high light can lead to PSI photoinhibition in leaves of shade-established tree species.

It has been shown that PSII activity can be quickly repaired under low light in several hours due to the fast turnover of D1 protein (Aro et al., 1993a,b; Allakhverdiev and Murata, 2004; Murata et al., 2007). In contrast to PSII activity, the recovery of PSI activity from photoinhibition is a slow process that needs several days (Kudoh and Sonoike, 2002; Zhang and Scheller, 2004). The fast recovery of PSII activity under low light is dependent on moderate PSI activity (Kudoh and Sonoike, 2002; Huang et al., 2010a,b; Tikkanen et al., 2010). Severe PSI photoinhibition depressed the rate of PSII repair or caused the failing of PSII recovery (Kudoh and Sonoike, 2002; Huang et al., 2010a). Since PSI complex involves in the activation of CEF (Peng and Shikanai, 2011), an irreversible PSI photoinhibition lead to a depression of CEF activity (Huang et al., 2013). The activation of CEF on condition of excess light energy is essential for PSII activity, impairment of CEF activity not only aggravated the rate of photodamage to PSII (Takahashi et al., 2009), but also increased the production of reactive oxygen species that inhibit the synthesis of D1 protein (Nishiyama et al., 2001, 2004, 2005, 2006, 2011; Takahashi et al., 2009). Thus, severe PSI photoinhibition can finally cause the failure of PSII recovery and finally the death of the plant (Huang et al., 2010a; Tikkanen et al., 2010). We speculate that the susceptibility to high light in leaves of shade-established tree species may be partly due to PSI photoinhibition.

In our present study, we examined the effect of high light on PSI and PSII activities in shade leaves of two tropical tree species *Psychotria rubra* (shade-established) and *Pometia tomentosa* (sun-established). Furthermore, the changes in P700 oxidation ratio and electron flow through PSII and PSI were determined during high light treatments. The following question was addressed: is PSI susceptible to high light in leaves of the shade-established species *P. rubra*?

## Materials and Methods

### Plant Materials and Growth Conditions

Two tropical tree species, *Psychotria rubra* (Lour.) Poir. (Rubiaceae) and *Pometia tomentosa* (Blume) Teijsm. & Binn (Sapindaceae) were studied in our present study. *P. rubra* is a shade-established shrub species of rain forests native to south of China, Indonesia, Vietnam, Laos, and Malaysia. *Pometia tomentosa* is a sun-established tree species and plants of this species can reach 45 m height. *P. tomentosa* is a dominant canopy species of tropical rain forests native to Sri Lanka, Indonesia, and southern Yunnan, China. They grown naturally in tropical rain forest in Xishuangbanna tropical botanical garden (21°54′ N, 101°46′ E) that is located in the northern boundary of tropical zone. Mature leaves of shade plants for both species grown at an understory environment with deep shade (light intensity < 5% of sunlight) were used for photosynthetic measurements.

### PSI and PSII Measurements

The PSI and PSII parameters were measured simultaneously by Dual-PAM-100 (Heinz Walz GmbH, Effeltrich, Germany) as described in Huang et al. (2010a,b) and Suorsa et al. (2012). After high light treatment at 1000 or 2000 μmol photons m^–2^ s^–1^, Y(I), Y(II), and Y(ND) were measured after 3 min adaptation at 1033 or 1953 μmol photons m^–2^ s^–1^, respectively. During recovery at 100 μmol photons m^–2^ s^–1^, Y(I), Y(II), and Y(ND) were measured after 3 min adaptation at 100 μmol photons m^–2^ s^–1^. All photosynthetic measurements were conducted at 25°C.

In our present study, the maximum chlorophyll fluorescence after dark adaptation (*F_m_*) and maximum quantum yield of PSII [*F_v_*/*F_m_* = (*F_m_* – *F_o_*)/*F_m_*] were measured to estimate the PSII activity, this method has been used widely in previous studies (Suorsa et al., 2012; Huang et al., 2013; Tikkanen et al., 2014). *F_o_* was measured after 20 min dark adaptation without saturating pulse (Huang et al., 2013, 2015). *F_m_* was measured after 20 min dark adaptation upon illumination of a pulse (300 ms) of saturating light (10,000 μmol photons m^–2^ s^–1^). The effective quantum yield of PSII was calculated as Y(II) = (*F_m_*′ – *F_s_*)/*F_m_*′ (Genty et al., 1989), where *F_m_*′ is the maximum fluorescence after light adaptation and measured upon illumination of a saturating pulse (300 ms and 10,000 μmol photons m^–2^ s^–1^), *F_s_* is the steady-state fluorescence after light adaptation. The maximum photo-oxidizable P700 (*P_m_*) was recorded to estimate the amount of photo-oxidizable PSI centers (Huang et al., 2010a,b, 2013; Suorsa et al., 2012; Tikkanen et al., 2014). In our present study, *P_m_* was measured after 20 min dark adaptation. The P700^+^ signals (*P*) may vary between a minimal (P700 fully reduced) and a maximal level (P700 fully oxidized). The maximum level (*P_m_*) was determined with application of a saturation pulse (300 ms and 10,000 μmol photons m^–2^ s^–1^) after pre-illumination with far-red light. *P_m_*′ was determined similar to *P_m_* but with actinic light instead of far-red light. The quantum yield of PSI was calculated as Y(I) = (*P_m_*′ – *P*)/*P_m_*, and the P700 oxidation ratio was calculated as Y(ND) = *P*/*P_m_* (Huang et al., 2013, 2015; Tikkanen et al., 2014). The electron flow from PSII to PSI was estimated by ETR II = Y(II) × PPFD × 0.5 × abs I, where 0.5 is the proportion of absorbed light reaching PSI or PSII, and abs I is absorbed irradiance taken as 0.85 of incident irradiance. The electron flow through PSI was estimated by ETR I = Y(I) × PPFD × 0.5 × 0.85. Because we did not measure the accurate leaf absorbance and the proportion of absorbed light reaching PSI or PSII in a cross-sectioned leaf, the estimated ETRI and ETRII were not the absolute ETRI and ETRII.

To measure the light response changes in ETRI, ETRII, intact mature leaves were light adapted at a high light (1033 μmol photons m^–2^ s^–1^) for 15 min. Afterward, photosynthetic parameters were evaluated at 2-min intervals at photosynthetic photon flux densities (PPFDs) of 1957, 1599, 1292, 830, 536, 344, 221, 100, 42, and 11 μmol photons m^–2^ s^–1^.

### Photoinhibitory Treatments and Subsequent Recovery

To determine the response of PSI and PSII to high light, detached leaves of both species were exposed to 1000 or 2000 μmol photons m^–2^ s^–1^ at 25°C for 5 h. After exposure to 1000 or 2000 μmol photons m^–2^ s^–1^ for 2 h, leaves were illuminated at a low light of 100 μmol photons m^–2^ s^–1^ for recovery.

### Statistical Analysis

The results were displayed as mean values of six independent experiments. The data were subjected to analysis of variance (ANOVA) using the SPSS 16.0 statistical software. Tukey’s multiple comparison test was used at α = 0.05 significance level to determine whether significant differences exist among different treatments.

## Results

**FIGURE 1:**
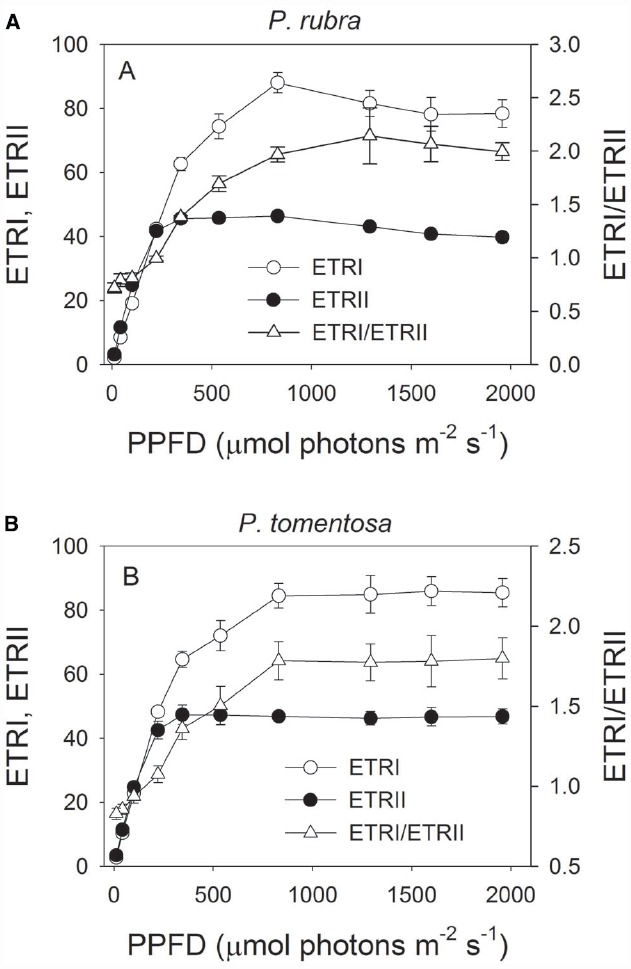
**Light response changes in photosynthetic electron flow through PSI and PSII (ETRI and ETRII), and ETRI/ETRII ratio in leaves of ***Psychotria rubra*** (A) and ***Pometia tomentosa*** (B) measured at 25°C.** The means ± SE were calculated from six independent plants. The effect of light intensity on the ETRI/ETRII ratio was analyzed by one-way ANOVA. Statistical analysis results indicated that cyclic electron flow was significantly activated under high light in both species (*P* < 0.05, One-way ANOVA).

Light response curves indicated that ETRI was largely higher than ETRII in both species when illuminated at light intensities above 344 μmol photons m^–2^ s^–1^ (Figures 1A,B). The ETRI/ETRII ratio under high light was much higher than that under low light in both species (Figures 1A,B). For example, at 11 μmol photons m^–2^ s^–1^, ETRI/ETRII ratio was 0.72 and 0.83 in *P. rubra* and *P. tomentosa*, respectively. At 1957 μmol photons m^–2^ s^–1^, ETRI/ETRII ratio was 2.0 and 1.8 in *P. rubra* and *P. tomentosa*, respectively. Because the activation of CEF leads to the increase in ETRI/ETRII ratio (Yamori et al., 2011; Kono et al., 2014; Huang et al., 2015), our results suggested that both species showed highly activation of CEF under high light.

**FIGURE 2:**
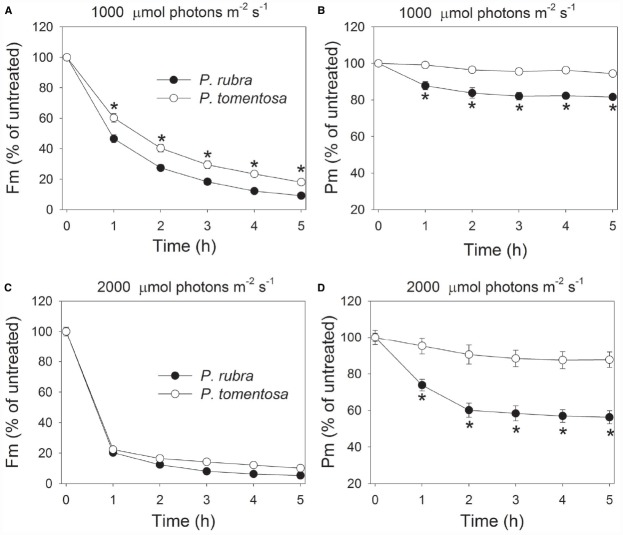
**Changes in ***F_m_*** (A,C) and ***P_m_*** (B,D) in ***Psychotria rubra*** and ***Pometia tomentosa*** during treatments at 25°C associated with high light intensities of 1000 (A,B) and 2000 (C,D) μmol photons m^–2^ s^–1^.** The means ± SE were calculated from six independent plants. Asterisks indicate significant differences between *Psychotria rubra* and *Pometia tomentosa*.

In order to examine the effect of high light on PSI and PSII in both species, detached leaves of *P. rubra* and *P. tomentosa* were exposed to light intensities of 1000 or 2000 μmol photons m^–2^ s^–1^. After exposure to 1000 μmol photons m^–2^ s^–1^ for 3 h, *F_m_* decreased by 82 and 70% in *P. rubra* and *P. tomentosa*, respectively (Figure 2A). Meanwhile, *P_m_* decreased by 18 and 4% in *P. rubra* and *P. tomentosa*, respectively (Figure 2B). When treated at 2000 μmol photons m^–2^ s^–1^ for 2 h, *P_m_* decreased by 40 and 10% in *P. rubra* and *P. tomentosa*, respectively, and *F_m_* decreased by 88% (*P. rubra*) and 83% (*P. tomentosa*; Figures 2C,D). These results indicated the shade-established species *P. rubra* showed higher sensitivity to PSI photoinhibition under high light. The susceptibility of PSI activity to high light in *P. rubra* was dependent on the intensity of illumination. Interestingly, after exposure to 1000 μmol photons m^–2^ s^–1^ for 3 h or 2000 μmol photons m^–2^ s^–1^ for 2 h, PSI activity decreased very slightly during further high light treatment in *P. rubra* (Figures 2B,D).

**FIGURE 3:**
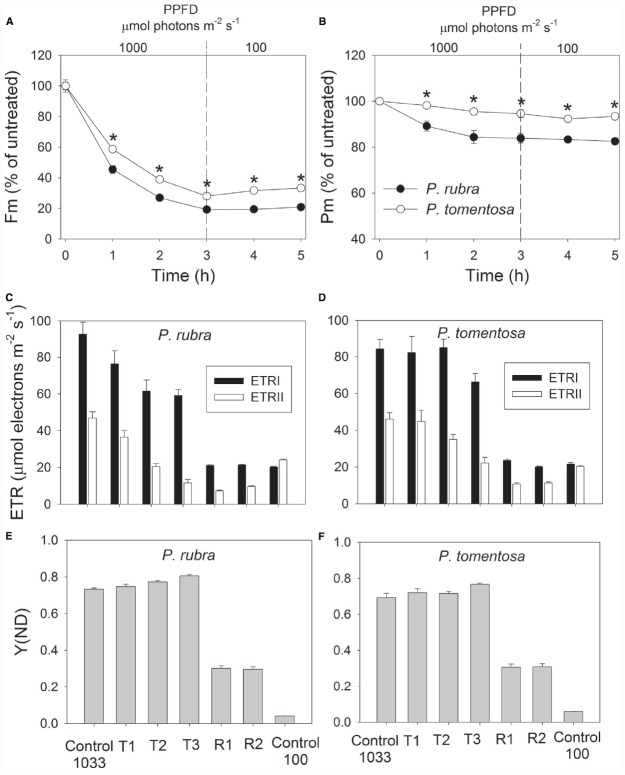
**Changes in ***F_m_*** (A), ***P_m_*** (B), ETR (C,D), and Y(ND) (E,F) in ***Psychotria rubra*** and ***Pometia tomentosa*** during treatment at 25°C and 1000 μmol photons m^–2^ s^–1^ and subsequent recovery at 100 μmol photons m^–2^ s^–1^.** T1, T2, and T3 represent the data after high light treatment for 1, 2, and 3 h, respectively. R1 and R2 represent the data after recovery at low light for 1 and 2 h, respectively. Control 1033 and Control 100 represent the data measured at 1033 and 100 μmol photons m^–2^ s^–1^ before treatment, respectively. The means ± SE were calculated from six independent plants. Asterisks indicate significant differences between *Psychotria rubra* and *Pometia tomentosa*.

**FIGURE 4:**
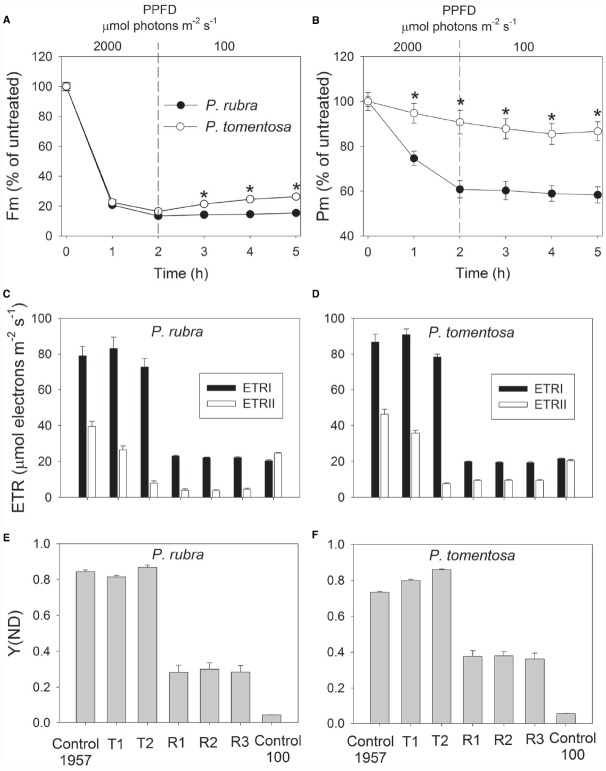
**Changes in Fm (A), Pm (B), ETR (C,D), and Y(ND) (E,F) in ***Psychotria rubra*** and ***Pometia tomentosa*** during treatment at 25°C and 2000 μmol photons m^–2^ s^–1^ and subsequent recovery at 100 μmol photons m^–2^ s^–1^.** T1 and T2 represent the data after high light treatment for 1 and 2 h, respectively. R1, R2, and R3 represent the data after recovery at low light for 1, 2, and 3 h, respectively. Control 1957 and Control 100 represent the data measured at 1957 and 100 μmol photons m^–2^ s^–1^ before treatment, respectively. The means ± SE were calculated from six independent plants. Asterisks indicate significant differences between *Psychotria rubra* and *Pometia tomentosa*.

After high light treatment at 1000 or 2000 μmol photons m^–2^ s^–1^, the detached leaves of both species were illuminated at 100 μmol photons m^–2^ s^–1^ for recovery. The value of *P_m_* changed slightly during the recovery process in *P. rubra* (Figures 3B and 4B), which further confirmed the high-light-induced PSI photoinhibition. For the sun-established species *P. tomentosa*, PSI was insusceptible to high light. *F_m_* increased slowly during recovery at 100 μmol photons m^–2^ s^–1^ (Figures 3A and 4A).

With increasing time of illumination at 1000 or 2000 μmol photons m^–2^ s^–1^, PSII photoinhibition gradually increased, and ETRII gradually decreased in both species. After treatment with 1000 μmol photons m^–2^ s^–1^ for 3 h, ETRII decreased by 75% in *P. rubra* (from 46.9 to 11.6 μmol electrons m^–2^ s^–1^), and by 52% in *P. tomentosa* (from 46.0 to 22.2 μmol electrons m^–2^ s^–1^) (Figures 3C,D). After treatment with 2000 μmol photons m^–2^ s^–1^ for 2 h, ETRII decreased by 80 and 82% in *P. rubra*, and *P. tomentosa*, respectively (Figures 4C,D). These results suggested that high levels of PSII photoinhibition depressed electron transfer from PSII to PSI. During the high light treatment, ETRII decreased faster than ETRI in both species (Figures 3C,D and 4C,D). As a result, the values for ETRI—ETRII and ETRI/ETRII increased during high light treatment. Furthermore, the P700 oxidation ratio was maintained at high levels during high light treatment in both species, as shown by the data of Y(ND; Figures 3E,F and 4E,F). These results indicated that CEF was highly activated during exposure to 1000 or 2000 μmol photons m^–2^ s^–1^ in both species.

During recovery at 100 μmol photons m^–2^ s^–1^, ETRI changed slightly compared with the original values in both species, but ETRII largely declined. After treatment at 1000 μmol photons m^–2^ s^–1^ for 3 h, values for ETRII at 100 μmol photons m^–2^ s^–1^ decreased to 30 and 52% of the original values in *P. rubra* and *P. tomentosa*, respectively (Figures 3C,D). After exposure to 2000 μmol photons m^–2^ s^–1^ for 2 h, values for ETRII during recovery were approximately 17 and 46% of the original values in *P. rubra* and *P. tomentosa*, respectively (Figures 4C,D). Meanwhile, the value of Y(ND) at 100 μmol photons m^–2^ s^–1^ largely increased during the recovery compared the original values (Figures 3E,F and 4E,F). This result indicated that CEF was also highly activated during recovery at low light in both species. The decline in ETRII and activation of CEF during recovery induced the increase in Y(ND).

**FIGURE 5:**
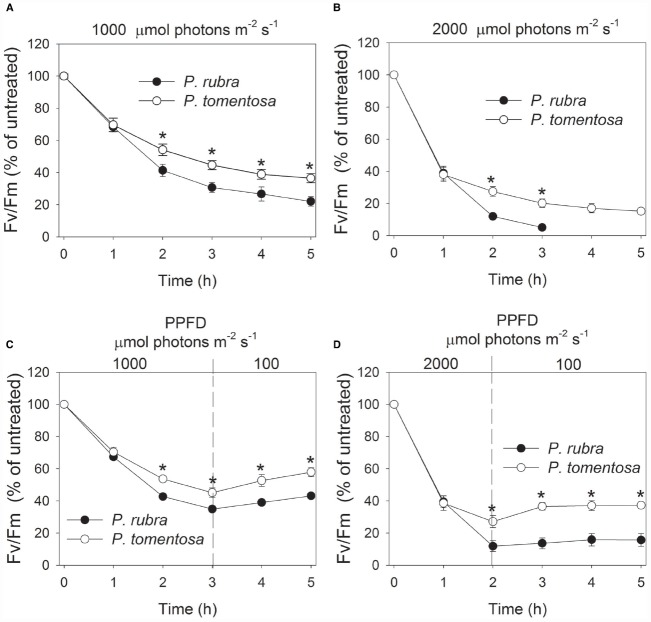
**Changes in ***F_v_***/***F_m_*** in ***Psychotria rubra*** and ***Pometia tomentosa*** during treatments at 25°C associated with high light intensities of 1000 (A) and 2000 (B) μmol photons m^–2^ s^–1^ and subsequent recovery at 100 μmol photons m^–2^ s^–1^ (C,D).** The means ± SE were calculated from six independent plants. Asterisks indicate significant differences between *Psychotria rubra* and *Pometia tomentosa*.

During the photoinhibitory treatment at 1000 and 2000 μmol photons m^–2^ s^–1^, *F_v_*/*F_m_* gradually decreased in both species (Figures 5A,B). After treatment for 2 h and more time, the decrease in *F_v_*/*F_m_* was larger in *P. rubra* than *P. tomentosa*. Interestingly, *F_v_*/*F_m_* decreased to 5% of the original value in *P. rubra* after treatment at 2000 μmol photons m^–2^ s^–1^ for 3 h. These results indicated that high light induced stronger PSII photoinhibition in *P. rubra*. After exposure to 1000 μmol photons m^–2^ s^–1^ for 3 h, *F_v_*/*F_m_* gradually increased during recovery at 100 μmol photons m^–2^ s^–1^ in both species (Figure 5C). However, *F_v_*/*F_m_* hardly increased during recovery in *P. rubra* following treatment at 2000 μmol photons m^–2^ s^–1^ for 2 h (Figure 5D).

**FIGURE 6:**
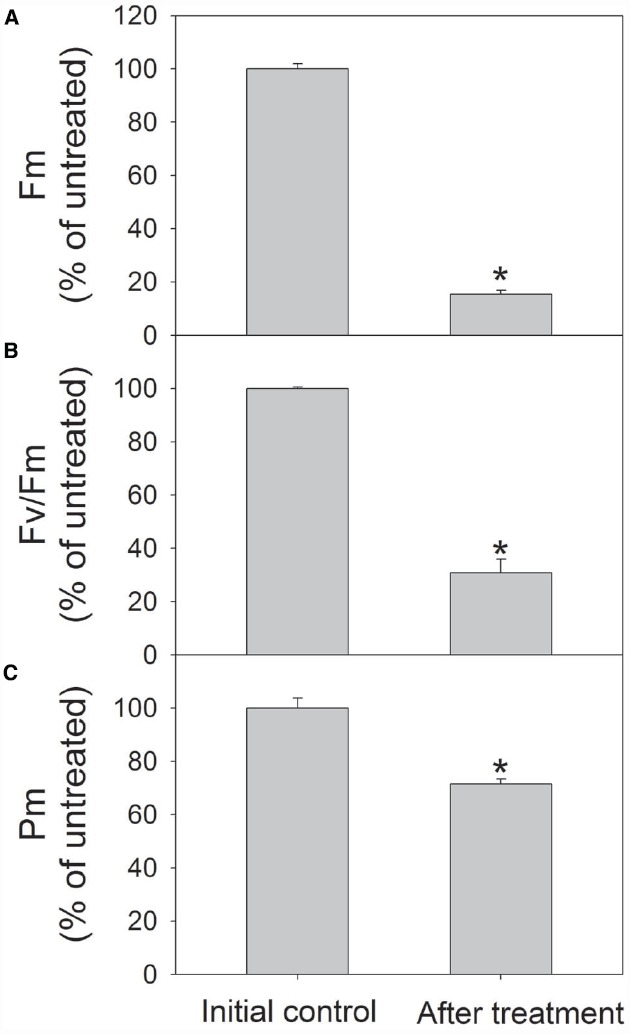
**Changes in ***F_m_*** (A), ***F_v_***/***F_m_*** (B), and ***P_m_*** (C) in ***Pittosporopsis kerrii*** after exposure to 2000 μmol photons m^–2^ s^–1^ for 4 h.** The means ± SE were calculated from six independent plants. Asterisks indicate significant differences between initial control and after treatment.

To further confirm the susceptibility of PSI to high light in shade-established species, mature leaves of another shade-established species *Pittosporopsis kerrii* (Icacinaceae) were treated at high light. After exposure to 2000 μmol photons m^–2^ s^–1^ for 4 h, *F_m_* and *F_v_*/*F_m_* largely decreased as expectedly. Meanwhile, *P_m_* decreased significantly by 29% (Figure 6). These results suggested that PSI activity was susceptible to high light not only in *P. rubra*, but also in another shade-established species *P. kerrii*.

## Discussion

It has been often considered that high light induces selective photodamage to PSII in nature, excluding chilling stress. However, the response of PSI to high light for shade-established tree species in unclear. Here we provide evidence that the shade-established species *P. rubra* is incapable of protecting PSI under high light. In addition, another shade-established species *P. kerrii* showed significantly PSI photoinhibition after exposure to high light. These results suggested that PSI photoinhibition is another important photosynthetic mechanism underlying the sensitivity of shade-established species to high light.

In a previous study, PSI activity remaining unaffected in tropical shade leaves after exposure to 1800 μmol photons m^–2^ s^–1^ for 75 min (Barth et al., 2001). In our present study, PSI activity also changed little after exposure to 1000 or 2000 μmol photons m^–2^ s^–1^ for 2 h in shade leaves of the sun-established species *P. tomentosa* (Figures 2B,D). In sharp contrast to *P. tomentosa*, PSI activity in the shade-established species *P. rubra* decreased to 60% of the original value after exposure to 2000 μmol photons m^–2^ s^–1^ for 2 h (Figure 2D). These results indicate that the response of PSI activity to high light in shade leaves is dependent on the plant successional trait.

Photosystem I photoinhibition is mainly induced by the deleterious effect of hydroxyl radicals. Excess electrons transported from PSII to the acceptor side of PSI result in the formation of superoxide anion radicals as well as in the reduction of iron–sulfur centers in PSI (Sonoike et al., 1997; Sonoike, 2011). The dismutation of superoxide anion radicals produces hydroxyl peroxide, which reacts with reduced iron-sulfur centers to form hydroxyl radicals that immediately destroy the iron–sulfur centers (Sonoike, 2006). The *pgr5*-mutants of *Arabidopsis thaliana* display photoinhibition of PSI under high light due to excess electrons from PSII to PSI and over-reduction of PSI acceptor side. In the present study, *P. rubra* showed high levels of P700 oxidation ratio during high light treatment. Thus, photoinhibition of PSI under high light in *P. rubra* was mainly caused by the excess electron transfer from PSII to PSI, but not correlated to over-reduction of PSI acceptor side. During exposure to 2000 μmol photons m^–2^ s^–1^, PSI photoinhibition in *P. rubra* mainly occurred in the initial course of 2 h and longer treatment did not enhance PSI photodamage. *P. rubra* showed significant active electron flow from PSII to PSI (67% of the original value) after exposure to 2000 μmol photons m^–2^ s^–1^ for 1 h (Figure 4C). However, after 2 h of treatment at this high light, ETRII decreased to 20% of the original value (Figure 4C). These results indicated that, in the initial stage of exposure to 2000 μmol photons m^–2^ s^–1^, the active electron flow from PSII to PSI induced the accumulation of hydroxyl radicals in the acceptor side and subsequently led to photodamage to PSI in *P. rubra*. Although the sun-established species *P. tomentosa* showed high levels of electron transfer from PSII to PSI during the initial treatment at 2000 μmol photons m^–2^ s^–1^, PSI activity was maintained stable. Therefore, *P. tomentosa* maybe had higher ability to scavenge reactive oxygen species. Alternatively, the sensitivity of PSI to reactive oxygen species differed between shade- and sun-established species. In the near future, it is necessary to study the molecular mechanism underlying why PSI is sensitive to high light in leaves of the shade-established species *P. rubra*.

It is assumed that PSI gets damaged only when electron flow from PSII exceeds the capacity of PSI electron acceptors to cope with the electrons (Tikkanen and Aro, 2014). When illuminated at low light, PSI activity changed little in both the wild type and *pgr5* mutants of *Arabidopsis thaliana*. However, the *pgr5* mutants showed large decrease in PSI activity when exposed to high light for several hours (Munekage et al., 2002; Tikkanen et al., 2014). For the shade-established species *P. rubra*, PSI activity changed little after exposure to 100 μmol photons m^–2^ s^–1^ for 5 h (data not shown). The significant PSI photoinhibition in the initial 2 h of high light treatment suggested that electron flow from PSII to PSI largely exceed the capacity of PSI electron acceptors in *P. rubra*. After 2 h exposure to 2000 μmol photons m^–2^ s^–1^, electron flow from PSII to PSI largely declined. Subsequently, PSI activity was maintained stable during further high light treatment. The PSI activity was protected against further photodamage in *pgr5* mutants of *Arabidopsis thaliana* upon moderate PSII photoinhibition, due to the depression of electron flow from PSII to PSI and the increase in P700 oxidation ratio (Tikkanen et al., 2014). After exposure to 2000 μmol photons m^–2^ s^–1^ for 2 h, *F_m_* decreased by approximately 85% in *P. rubra* (Figure 4A), which was accompanied with a large decrease in ETRII and no further photodamage of PSI (Figure 3C). Taking together, these results indicated that severe PSII photoinhibition restricts electron transfer from PSII to PSI and then alleviated PSI photodamage under high light in *P. rubra*.

On condition of excess light, CEF is activated to protect PSI and PSII against photoinhibition (Munekage et al., 2002; Takahashi et al., 2009; Tikkanen et al., 2014). CEF not only increases the P700 oxidation ratio (Munekage et al., 2002, 2004; Suorsa et al., 2012; Tikkanen et al., 2014) but also controls electron transfer from PSII to PSI via Cyt b6f complex (Tikkanen and Aro, 2014). At a normal growth temperature of 25°C, it has been believed that wild-type plants are capable of protecting PSI under high light (Munekage et al., 2002, 2004; Suorsa et al., 2012; Tikkanen et al., 2014; Huang et al., 2015). However, our present study indicated that the shade-established species *P. rubra* was incapable of protecting PSI under high light, although it showed highly activation of CEF under high light. The *pgr5*-mutans showed PSI photoinhibition under high light mainly due to low P700 oxidation ratio. Surprisingly, photoinhibition of PSI under high light in *P. rubra* was accompanied with high levels of P700 oxidation ratio. Therefore, the mechanism of PSI photoinhibition probably differed between *P. rubra* and *Arabidopsis thaliana*, and CEF is unlikely to play a major role in photoprotection for PSI in *P. rubra*.

Surprisingly, PSI activity showed slightly decrease in both species during recovery at a low light of 100 μmol photons m^–2^ s^–1^. As reported previously, light harvesting complex I proteins have a slow turnover (Kudoh and Sonoike, 2002; Zhang and Scheller, 2004). Once PSI photoinhibition happened, the photodamaged PSI complex is completely degraded (Zhang and Scheller, 2004). After 8 h recovery at low light and 20°C, PSI activity recovered very slowly, and the amount of PSI on a leaf area basis remained low even after recovery for 1 week (Zhang and Scheller, 2004). Therefore, the little recovery of PSI activity in *P. rubra* and *P. tomentosa* may be as a result of the slow turnover.

## Conclusion

Our results indicated that the shade-established tree species *Psychotria rubra* was incapable of protecting PSI under high light. Furthermore, highly activation of CEF could not prevent PSI against photoinhibition under high light in *P. rubra*. The extent of PSI photoinhibition under high light in *P. rubra* was mainly controlled by the electron transfer from PSII to PSI. When the canopy gaps are created, the strong sunlight can induce severe photoinhibition of both PSI and PSII in shade-established plant species. These findings suggest that photoinhibition of PSI may be an important mechanism explaining why shade-established species cannot survive under high light.

### Conflict of Interest Statement

The authors declare that the research was conducted in the absence of any commercial or financial relationships that could be construed as a potential conflict of interest.
